# 3D quantitative myocardial perfusion imaging with hyperpolarized HP001(bis‐1,1‐(hydroxymethyl)‐[1‐^13^C]cyclopropane‐d8): Application of gradient echo and balanced SSFP sequences

**DOI:** 10.1002/mrm.30320

**Published:** 2024-09-30

**Authors:** Yupeng Zhao, Rie Beck Olin, Esben Søvsø Szocska Hansen, Christoffer Laustsen, Lars G. Hanson, Jan Henrik Ardenkjær‐Larsen

**Affiliations:** ^1^ Department of Health Technology Technical University of Denmark Kgs. Lyngby Denmark; ^2^ MR Research Centre Aarhus University Aarhus Denmark; ^3^ Danish Research Centre for Magnetic Resonance, Centre for Functional and Diagnostic Imaging and Research Copenhagen University Hospital Hvidovre Hvidovre Denmark

**Keywords:** balanced SSFP, gradient echo, hyperpolarized HP001, MRI, quantitative myocardial perfusion imaging

## Abstract

**Purpose:**

This study aims to show the viability of conducting three‐dimensional (3D) myocardial perfusion quantification covering the entire heart using both GRE and bSSFP sequences with hyperpolarized HP001.

**Methods:**

A GRE sequence and a bSSFP sequence, both with a stack‐of‐spirals readout, were designed and applied to three pigs. The images were reconstructed using 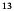
C coil sensitivity maps measured in a phantom experiment. Perfusion was quantified using a constrained decomposition method, and the estimated rest/stress perfusion values from 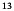
C GRE/bSSFP and Dynamic contrast‐enhanced MRI (DCE‐MRI) were individually analyzed through histograms and the mean perfusion values were compared with reference values obtained from PET(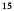
O‐water). The Myocardial Perfusion Reserve Index (MPRI) was estimated for 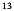
C GRE/bSSFP and DCE‐MRI and compared with the reference values.

**Results:**

Perfusion values, estimated by both DCE and 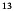
C MRI, were found to be lower than reference values. However, DCE‐MRI's estimated perfusion values were closer to the reference values than those obtained from 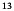
C MRI. In the case of MPRI estimation, the 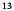
C estimated MPRI values (GRE/bSSFP: 2.3/2.0) more closely align with the literature value (around 3) than the DCE estimated MPRI value (1.6).

**Conclusion:**

This study demonstrated the feasibility of 3D whole‐heart myocardial perfusion quantification using hyperpolarized HP001 with both GRE and bSSFP sequences. The 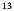
C perfusion measurements underestimated perfusion values compared to the 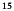
O PET literature value, while the 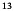
C estimated MPRI value aligned better with the literature. This preliminary result indicates 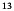
C imaging may more accurately estimate MPRI values compared to DCE‐MRI.

## 1 INTRODUCTION

Coronary artery disease is the most common heart disease and a leading cause of death globally.^1^ It develops when plaque build‐up inside coronary arteries reduces or blocks the blood supply to myocardial tissue.

Dynamic contrast‐enhanced MRI (DCE‐MRI) is commonly used clinically for coronary artery disease diagnosis.^2^ It employs gadolinium (Gd)‐based MR contrast agents and dynamic acquisition of a series of T_1_‐weighted images to assess tissue perfusion.^3^, ^4^, ^5^ Despite its widespread use, DCE‐MRI faces challenges. There are concerns about potential Gd retention in the body and environmental damage.^6^, ^7^, ^8^, ^9^ Regulatory agencies have taken actions in response. The European Medicines Agency issued a recommendation to suspend three widely used Gd‐based MR contrast agents, and the US Food & Drug Administration has required new class warnings for these agents. Additionally, DCE‐MRI is an indirect perfusion measurement method, potentially leading to inaccurate perfusion quantification values.^10^ Furthermore, image quality and resolution are limited by SNR.

As an alternative to Gd‐based contrast agents, hyperpolarized 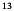
C‐based contrast agents can be used to assess myocardial perfusion. Some studies have shown the feasibility of performing two‐dimensional (2D) myocardial perfusion imaging using hyperpolarized 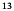
C urea and pyruvate with Gradient Echo Sequence (GRE) and Balanced Steady‐State Free Precession Sequence (bSSFP) type sequences.^11^, ^12^, ^13^, ^14^, ^15^, ^16^ Three‐dimensional (3D) techniques provide volumetric coverage of the entire heart, allowing for a more comprehensive assessment of myocardial perfusion in a single acquisition. Also, 3D techniques may offer better signal‐to‐noise ratio (SNR) compared to 2D techniques, potentially leading to higher image quality. However, 3D myocardial perfusion imaging is challenging due to the short acquisition window that limits spatial and temporal resolution.

GRE and bSSFP sequences are the most used sequences in hyperpolarized MRI. Both sequences have their pros and cons. GRE sequences benefit from their fast encoding speed, making them suitable for imaging fast‐decaying hyperpolarized contrast agents. However, the signal decays rapidly with an increasing flip angle. bSSFP sequences provide efficient use of the non‐recoverable magnetization in hyperpolarized 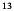
C MRI. However, the need for shorter pulse repetition time (TR) in bSSFP imposes limitations on the amount of k‐space data that can be acquired within a single TR, leading to a relatively slower recording. Additionally, bSSFP's susceptibility to off‐resonance further constrains its applicability.

HP001 (bis‐1,1‐(hydroxymethyl)‐[1‐^13^C]cyclopropane‐d8) is a synthesized metabolically inert compound with good properties for dynamic nuclear polarization methods. It has long T_1_ and T_2_ relaxation times due to its molecular structure and deuteration (T_1_/T_2_ are around 82/18 s at 2.35 T in a water solution).^17^ Therefore, it is suitable for myocardial perfusion measurements. Early studies have demonstrated methods for using HP001 in tissue perfusion in rats and mice.^18^, ^19^, ^20^

In this work, we present methodology for 3D whole‐heart myocardial perfusion quantification using hyperpolarized HP001 with both GRE‐ and bSSFP‐type sequences. The proposed imaging methods were tested in three healthy pigs. The ^13^C images and DCE‐MRI images were compared in terms of SNR. The perfusion values estimated by ^13^C GRE/bSSFP and DCE‐MRI were analyzed through histograms and compared.

## 2 METHODS

### 2.1 Sequence design and simulation

**FIGURE 1:**
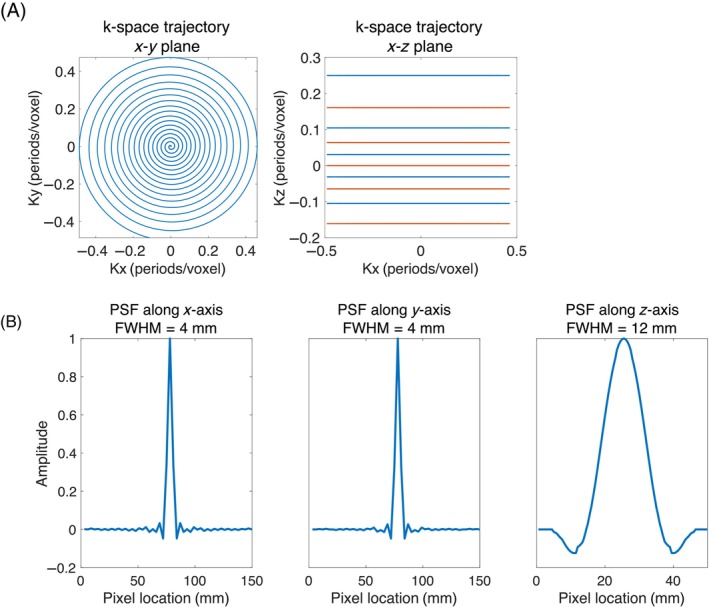
(A) Stack‐of‐spiral k‐space sampling trajectories for the GRE sequence. The odd‐numbered planes (red lines) were acquired first, followed by the even‐numbered planes (blue lines) to enable sliding window reconstruction. (B) The point spread function of the designed GRE sequence. The full width at half maximum (FWHM) reflects the actual resolution, which is $3\times 3\times 12\;{\text{mm}}^{3}$.

A spoiled GRE sequence with a 3D center‐out variable density stack‐of‐spirals readout was designed (Figure 1A). The sampling density from the center to the outer k‐space was linearly reduced. This variable density sampling strategy reduces image ringing artifacts.^21^ The sequence parameters are as follows: field of view (FOV) = $150\times 150\times 100\;{\text{mm}}^{3}$, resolution $=3\times 3\times 10\;{\text{mm}}^{3}$, readout time = 21 ms, flip angle $=10^{\circ }$, TR/echo time(TE) = 44/1 ms. To enable sliding window reconstruction, the data acquisition order for k_z_ phase encoding was chosen to first acquire odd‐numbered planes and then even‐numbered planes. The spatial point spread function (PSF) was simulated by performing a nonuniform Fourier transform with all k‐space data points set to ones (Figure 1B), thus ignoring radiofrequency (RF) pulsing and relaxation effects considering the long T_1_ relaxation time (approximately 30 s in blood) of hyperpolarized HP001 compared to the single‐frame image acquisition time (around 400 ms) and the small number of RF pulses (10 excitations) applied in a single k‐space acquisition.

**FIGURE 2:**
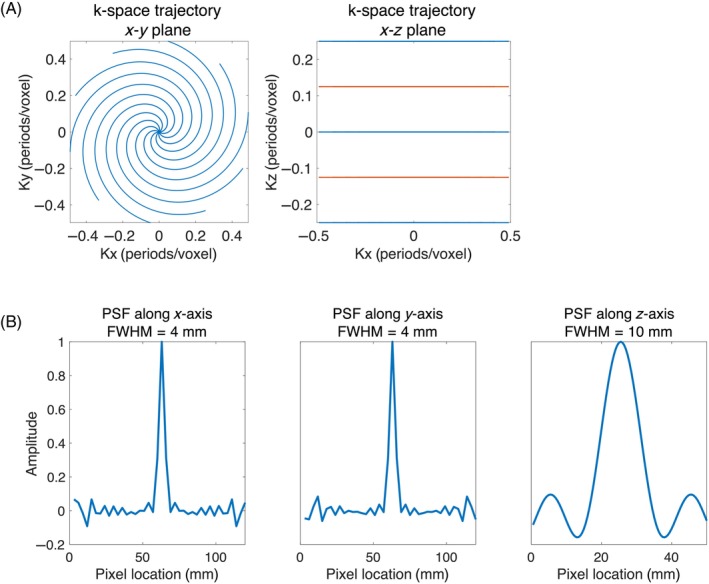
(A) Stack‐of‐spiral k‐space sampling trajectory for the bSSFP sequence. Similar to the GRE sequence, the odd‐numbered planes (red color) and even‐numbered planes (blue color) were separately acquired to enable the sliding window approach. (B) The point spread function of the designed bSSFP sequence.

The bSSFP sequence included slice‐selective excitations and 3D stack‐of‐spirals readouts (Figure 2A). To shorten the TR, the spiral trajectory is undersampled by a factor of two, resulting in each spiral interleave having a 3 ms readout time. The stack consisted of five planes, and each plane contained 10 interleaved spiral readouts. All gradients were balanced during each TR. The in‐plane k‐space sampling density was linearly reduced from the center to the outer k‐space.^21^ Initially, the k_z_ phase encoding was chosen with a suboptimal variable density that was applied in the first pig experiment. However, this approach led to a PSF with excessively large sidebands. To mitigate this issue, the k‐space planes were subsequently redesigned with uniform density sampling, as shown in Figure 2A. The sequence parameters are: FOV = $120\times 120\times 50\;{\text{mm}}^{3}$, resolution = $3\times 3\times 10\;{\text{mm}}^{3}$, TR/TE = 7/2 ms, flip angle = 40°, and the RF phase is alternating. For both 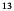
C bSSFP and GRE sequence designs, the MNS Research Pack is used (software for sequence development, supplied by GE Healthcare).

Ten linear ramp‐up pulses with increasing flip angles were used as magnetization preparation to approximately enter pseudo‐steady‐state and therefore reduce transient signal fluctuations. The same pulses in reverse order were used as flip‐back pulses. The flip‐back pulses brings the magnetization back to longitudinal to save it during the waiting time. The RF phase was alternating to center a passband on resonance.

The mathematical formulation to obtain the steady‐state magnetization **M_ss_** is well‐known for thermal MRI.^22^ We reformulated the equations for the hyperpolarized case. The magnetization propagation from one TR to the next can be described by a product of two matrices **R** and **D**. The rotation matrix **R** describes the excitation with flip angle θ. Matrix **D** describes the off‐resonance rotation by angle β (including phase‐cycling), and the T_1_ and T_2_ relaxation that happens during one TR. Unlike in thermal MRI, the T_1_ relaxation results in signal decay instead of recovery. The transformation matrix T=DR propagates the magnetization in one TR period.

(1) $$R=\left[\begin{array}{lll} 1 & 0 & 0\\ 0 & \cos\theta & \sin\theta \\ 0 & -\sin\theta & \cos\theta \end{array}\right].$$

(2) $$D=\left[\begin{array}{lll} e^{-\frac{\text{TR}}{T_{2}}}\cos\beta & e^{-\frac{\text{TR}}{T_{2}}}\sin\beta & 0\\ -\;e^{-\frac{\text{TR}}{T_{2}}}\sin\beta & e^{-\frac{\text{TR}}{T_{2}}}\cos\beta & 0\\ 0 & 0 & e^{-\frac{\text{TR}}{T_{1}}} \end{array}\right].$$

The pseudo‐steady‐state magnetization **M_ss_** is calculated as an eigenstate from the equation below, where δ is the eigenvalue, with its value ranging between 0 and 1, describing signal decay per TR.

(3) $${T\;M}_{\text{ss}}=\delta \;M_{\text{ss}}.$$

Using this method, the pseudo‐steady‐state magnetization **M_ss_** and transverse magnetization evolution were simulated for a range of off‐resonance phase between ($-180^{\circ }$, 180°]. The simulated sequence parameters were chosen to match those used in vivo. The T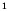 and T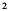 relaxation times were set to the HP001 T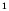 and T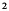 relaxation times measured in the blood.^17^

### 2.2 Image reconstruction

A sliding window approach^23^ was employed and is briefly explained here. In both 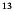
C GRE and bSSFP sequences, the acquisition of the k‐space is divided into separate acquisition of two subsets, each containing either even‐ or odd‐numbered phase encodes. During the reconstruction, these incompletely sampled subsets are combined to form a fully sampled dataset, from which a 3D image is subsequently reconstructed. After the initial full dataset is used to reconstruct an image, the process continues by replacing the oldest subset with the most recently acquired one, creating a new full dataset. This new dataset is then used to reconstruct the next image in the dynamic series. Before reconstruction, a filter was designed and applied to the variable‐density‐sampled k‐space data to match the k‐space density function to a Gaussian function, reducing image ringing artifacts. The filter was calculated using a truncated Gaussian function divided by the smoothed k‐space density function. The k‐space sampling density function was visualized by performing a nonuniform Fourier transformation on an image representing a point source object. Conjugate gradient SENSE reconstruction was performed using the MIRT toolbox.^24^, ^25^

### 2.3 Coil sensitivity mapping

For the in vivo experiment, a home‐built 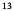
C eight‐channel flexible coil^26^ was used for reception and a clamshell‐type coil for transmission (RAPID Biomedical GmbH).

**FIGURE 3:**
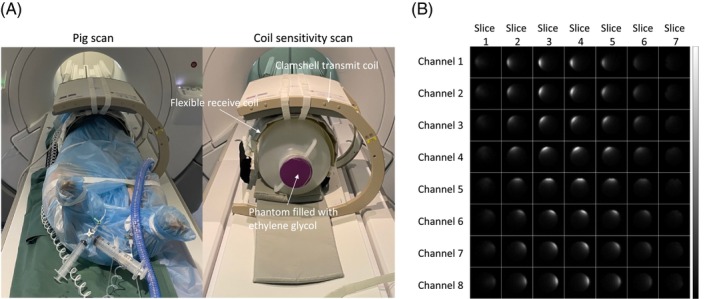
(A) The coil setup for both in vivo and phantom experiments. The shape of the flexible receive coil was placed on a homemade fiberglass mold, and its position was secured using Velcro straps. With this setup, the shape and position of the receive coil remained consistent between the phantom and in vivo experiments. (B) The measured sensitivity maps of the eight‐channel 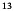
C receive coil using the configuration shown in Figure 3A.

Coil sensitivity maps are essential for SENSE reconstruction. The 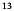
C coil sensitivity mapping procedure was adapted from that described in Reference 27, mainly because a different sequence and a flexible coil were used in the current study. First, an in vivo experiment was conducted while the receive coil's shape and location were fixed relative to the transmission coil, as shown in Figure 3A. The coil fixation procedure and the relatively low RF frequency ensure that the coil sensitivity profiles remain unchanged from the in vivo experiment to the phantom experiment. After the in vivo experiment, the pig was carefully replaced by an ethylene glycol phantom, made as described in Reference 27. To emulate tissue loading, 17 g/L of NaCl (291 mmol/L 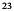
Na concentration) is added to the phantom. Hence, the coil sensitivity map was measured and processed with the protocol described below:

- A 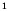
H B_0_ map was acquired using a multislice multi‐echo spoiled GRE sequence. The sequence parameters are as follows: FOV = 260 × 260 mm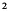, matrix size = 128 × 128, TE = 4.7 ms, TR = 100 ms, number of echoes = 8, number of slices = 20, slice thickness = 10 mm, flip angle = 30°.
- The 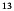
C coil sensitivity mapping was performed using a multislice 2D spiral spoiled GRE sequence with spectral‐spatial excitation. The sequence parameters are as follows: FOV=400×400 mm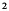, matrix size = 20×20, readout time = 27 ms, TR = 1000 ms, number of slices = 13, slice thickness = 20 mm, flip angle = 70°, number of averages = 1500.
- The B_1_+ mapping was based on the Bloch–Siegert shift and used a multislice 2D spiral Bloch–Siegert spoiled GRE with spectral‐spatial excitation. The sequence was described in Reference 28. The sequence parameters are the same as in the sensitivity scan.

In the sensitivity mapping scan, a spectral and spatially selective RF pulse was used. In the presence of significant off‐resonance (exceeding half the RF spectral bandwidth), the excited signal was reduced. To address this issue, the sensitivity maps were corrected based on the measured B_0_ maps and RF spectral profile, compensating for the reduced signal due to off‐resonance. Relative B_1_+ maps were acquired and used to compensate flip angle variations. Finally, the coil sensitivity maps were registered to the 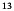
C in vivo images based on the scanner coordinates. Figure 3B depicts the measured coil sensitivity maps.

### 2.4 In vivo experiment

#### 2.4.1 Animal handling

The animal experiments were approved by the Danish Animal Inspectorate. Three healthy 40 kg female Danish domestic pigs participated in the study. The pigs were presedated, and anesthesia was maintained by continuous intravenous infusion of Propofol. The pigs were intubated and mechanically ventilated. Amiodarone was used before the experiment to stabilize the heartbeat.

#### 2.4.2 Hyperpolarization

HP001 (GE Healthcare) was hyperpolarized using the dissolution dynamic nuclear polarization method. For each injection, a mixture of 300 μL HP001 with a 40 mmol/L trityl radical (AH111501, GE Healthcare) was polarized with a SpinAligner polarizer (Polarize ApS) for around 90 min and dissolved into 15 mL saline. The 40 mmol/L radical concentration was optimized to achieve an acceptable build‐up time for the in vivo experiment without a significant loss of polarization. The HP001 concentration was approximately 9.7 mol/L in the sample and 166 mmol/L in the dissolved solution. After dissolution, the sample was aspirated into a syringe and immediately transported to the MR‐scanner room for injection. A small fraction of the sample (500 μL) was taken aside and used for measuring the polarization and T_1_ relaxation time.

#### 2.4.3 Imaging protocol

All imaging experiments were performed in a 3T scanner (Discovery MR750, GE Healthcare). For each in vivo experiment, rest and stress perfusion imaging with 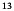
C GRE/bSSFP and DCE were performed. A total of four hyperpolarized HP001 and two gadolinium (DOTAREM [Guerbet]) injections were manually administrated for each pig. The injection volume of DOTAREM was determined based on the recommended dose, which is 0.2 mL kg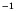. Stress was induced by continuous infusion of a mixture of adenosine (300 μg/min/kg) and dobutamine (15 μg/min/kg)(Pfizer). The imaging started 5 min after the beginning of infusion. The imaging protocol:

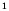
 **H multiphase anatomical images**: Acquired in a short axis view using a multislice 2D cine FIESTA (steady‐state free precession type sequence) with breath hold. Sequence parameters: FOV = 400 × 400 mm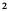, resolution = 0.78 × 0.78 mm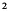, TR/TE = 3.4/1.5 ms, slice thickness = 10 mm, flip angle = 55°, cardiac phases = 30.
- **RF transmission gain (TG) and** 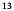 **C HP001 center frequency calibration**: A gadolinium‐doped 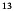
C‐bicarbonate phantom was placed over the pig chest and used for calibrating TG and center frequency. The calibration procedure followed an automated Bloch‐Siegert shift method described in Reference 29. After calibration, the 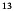
C HP001 center frequency was calculated from the calibrated 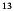
C‐bicarbonate frequency based on the chemical shift difference between the two compounds.
- **Rest/stress** 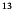 **C GRE perfusion images**: Acquired with cardiac gating in the diastole. For each cardiac trigger, a complete set of k‐space data was acquired (10 excitations). Sequence parameters: FOV = 150×150×100 ${\text{mm}}^{3}$, resolution = 3×3×10 ${\text{mm}}^{3}$, TR/TE = 44/1 ms, flip angle = 10°, image frame time = half the cardiac cycle.
- **Rest/stress** 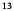 **C bSSFP perfusion images**: Acquired with cardiac gating in the diastole. For each cardiac trigger, a complete k‐space dataset was acquired (60 excitations). Sequence parameters: FOV = 120×120×50 ${\text{mm}}^{3}$, resolution = 3×3×10 ${\text{mm}}^{3}$, TR/TE = 7/2 ms, flip angle = 40°, image frame time = half a cardiac cycle.
- **Rest/stress** 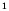 **H saturation‐recovery DCE‐MRI**: Performed with diastolic cardiac gating. Sequence parameters: FOV = 350×350 mm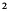, resolution = 1.35×1.35 mm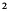, saturation time/TE/TR = 118/1.12/2.3 ms, flip angle = 20°, slice thickness = 8 mm, slice spacing = 20 mm, number of slices = 3, image frame time = 1 cardiac cycle.

#### 2.4.4 Perfusion quantification

The ventricular blood pool signal is much higher than the myocardial tissue signal. Due to partial volume effects, the myocardial signal is contaminated by the neighboring ventricular blood pool signal. To reduce perfusion quantification errors caused by partial volume effects, the constrained decomposition method^30^ was used for perfusion quantification for both 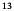
C and DCE data.

Signal saturation is a well‐known effect in DCE‐MRI and was observed in the DCE dataset. To reduce perfusion quantification errors caused by signal saturation, we adopt the following approaches for estimating perfusion from DCE data. First, we use the constrained decomposition method^30^ to estimate component weight maps and signal time courses. Then, we employ a method^31^ that utilizes only the upslope of the arterial input function and tissue signal to calculate perfusion. This method is, in principle, insensitive to the signal saturation effect. One important aspect of the upslope method is selecting the duration of the period used in the analysis. The chosen period should accurately describe the linear relationship expressed in equation (3) in Reference 31. To estimate the two unknowns, perfusion F and the initial rate at which the contrast agent leaves a voxel, at least two time course points are required. To increase the accuracy and robustness of the estimation against noise, our approach selects the first three points from the upslopes of arterial input function and tissue signal curves.

Due to computer memory limitations (20 cores with 4 GB of memory per core), each 3D 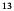
C dataset was randomly undersampled to a smaller subset containing approximately 400 voxels processed using the constrained decomposition method. The component time courses were then estimated from the subset, and the corresponding component weight maps were calculated by projection, that is, by multiplying the pseudo‐inverse of the matrix H, which contains the estimated component time courses, with the data matrix X, which contains 3D imaging data.

#### 2.4.5 Comparison of C and DCE‐MRI perfusion estimates

The perfusion values estimated from 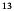
C GRE/bSSFP and DCE‐MRI were compared. Despite efforts to gate both the 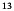
C and DCE sequences to diastole, the 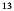
C GRE/bSSFP and DCE images were acquired in different cardiac phases due to changes in heart rate before and after the injection and the differences in acquisition times between the 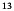
C and DCE sequences. Moreover the spatial PSFs differ. Therefore, the 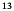
C and DCE perfusion maps were spatially mismatched. This made the spatial correlation of the perfusion values between 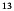
C GRE/bSSFP and DCE‐MRI impossible. Instead, histograms of rest/stress myocardial perfusion values from 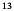
C GRE/bSSFP and DCE‐MRI were individually examined and compared. For each imaging method (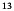
C GRE/bSSFP and DCE‐MRI), the Myocardial Perfusion Reserve Index (MPRI) was calculated for individual pigs by dividing the mean stress values by the mean rest values. MPRI serves as an important metric,^32^ reflecting the coronary arteries' ability to enhance blood supply to myocardial tissue during stress. By estimating MPRI values, one can compare the relative changes in perfusion between rest and stress conditions for 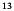
C GRE/bSSFP and DCE‐MRI.

## 3 RESULTS

### 3.1 Simulation

**FIGURE 4:**
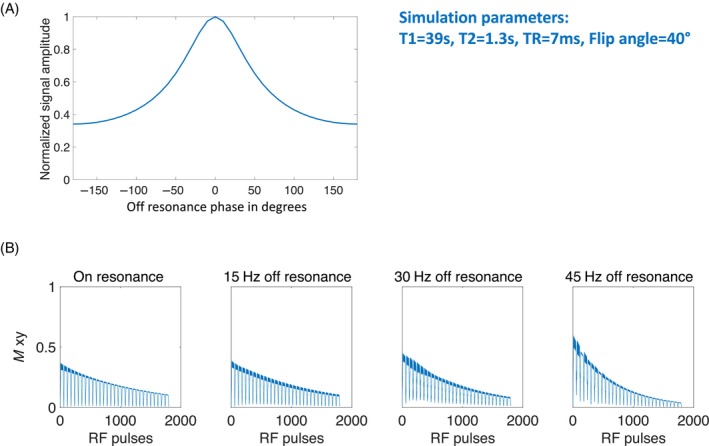
(A) The simulated pseudo‐steady‐state signal as a function of frequency offset $\beta -180^{\circ }$ for alternating radiofrequency (RF) excitation phases. (B) The signal evolution of the bSSFP sequence during an RF pulse train, simulated for four different off‐resonance values. The characteristic rapid signal drops and recoveries are due to the flip‐back and ramp‐up pulses. The sequence parameters used in vivo are applied, and the T_1_/T_2_ values in the simulation correspond to the measured HP001 T_1_/T_2_ values in blood.

The pseudo‐steady‐state signal was simulated for the bSSFP sequence with off‐resonance phases ranging between (−180 °, 180°] using the method introduced in the methodology section. Figure 4 displays the simulation results. The pseudo‐steady‐state signal decreases with increased off‐resonance phase. This signal behavior aligns with what is known for thermal MRI. Figure 4B illustrates the signal evolution of the bSSFP sequence. As observed, the signal decreases with increased number of excitations. The characteristic signal drops and jumps are the outcomes of ramp‐up and flip‐back pulses. With increasing off‐resonance phase, the signal tends to exhibit more oscillations and faster decay.

### 3.2 In Vivo Experiments

**FIGURE 5:**
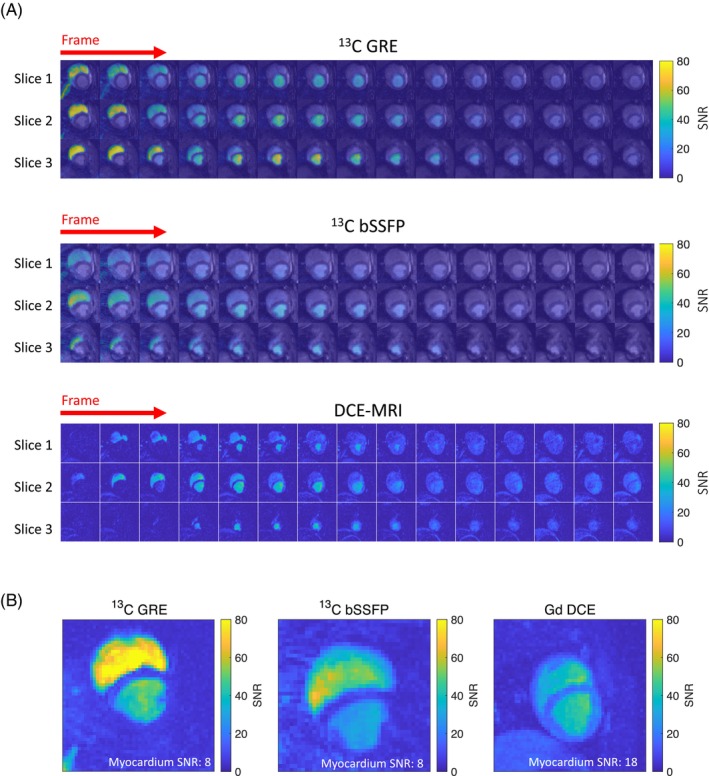
(A) Dynamic perfusion images acquired using 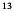
C GRE/bSSFP and dynamic contrast‐enhanced MRI (DCE‐MRI) presented in signal‐to‐noise ratio (SNR) units. The SD of the noise was estimated in a manually drawn background region free of visible hyperpolarized signal. Slices from the base, middle, and apex of the myocardium are displayed for each sequence, with all images acquired under stress conditions. (B) The maximum SNR maps of the middle slice, calculated as the ratio of the signal to the standard deviation of noise. For each voxel, the signal is determined as the maximum signal along the time dimension. For 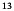
C GRE and bSSFP images shown in the figure, the polarization level measured at the time of injection is approximately 23%. It is evident from the figure that 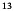
C exhibits higher right ventricle SNR but lower myocardium SNR compared to DCE‐MRI.

For all in vivo experiments, the measured liquid‐state HP001 polarization ranged between 20% and 40% at the time of injection, while the measured T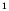 relaxation time at 1 T and approximately 28°C ranged from 65 to 75 s. 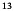
C GRE/bSSFP and DCE dynamic perfusion images are presented in Figure 5A in SNR units, acquired under stress conditions. Three slices from the base, middle, and apex of the myocardium are chosen for display. The images reveal the contrast arrival at the right ventricle followed by wash‐out, succeeded by the arrival at the left ventricle followed by wash‐out. However, the myocardial perfusion signal is not prominently visible due to its low amplitude compared to the left ventricular blood pool signal. The first three frames of the bSSFP images suffer from image ringing artifacts possibly stemming from aliasing of the first arriving signals outside the FOV. Figure 5B shows the maximum SNR maps of slice 2 shown in Figure 5A. Comparing the maximum SNR maps of 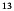
C and DCE, the 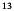
C images have higher SNR in the right ventricle and lower SNR in the myocardium compared to the DCE image. The myocardium is clearly visible in the DCE image but not in the 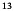
C SNR images. Both 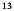
C GRE and bSSFP images exhibit similar left ventricle and low myocardium SNR.

**FIGURE 6:**
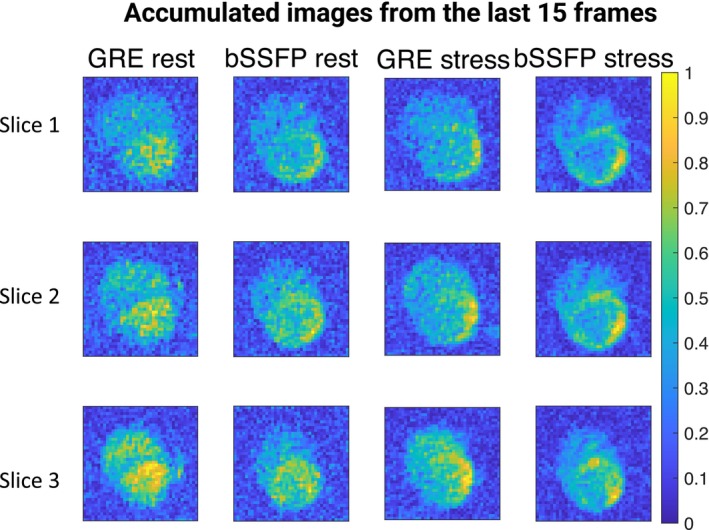
The accumulated rest/stress images from the last 15 frames of 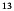
C bSSFP/GRE. Notably, bSSFP exhibits enhanced myocardium visibility compared to GRE in both rest and stress conditions.

Figure 6 displays accumulated images from the last 15 frames of rest/stress 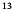
C GRE/bSSFP imaging. It reveals that the myocardium is visible in bSSFP images but not in GRE images. bSSFP images exhibit higher myocardium contrast than GRE images, potentially caused by a higher myocardial perfusion‐weighted signal in bSSFP compared to GRE.

**FIGURE 7:**
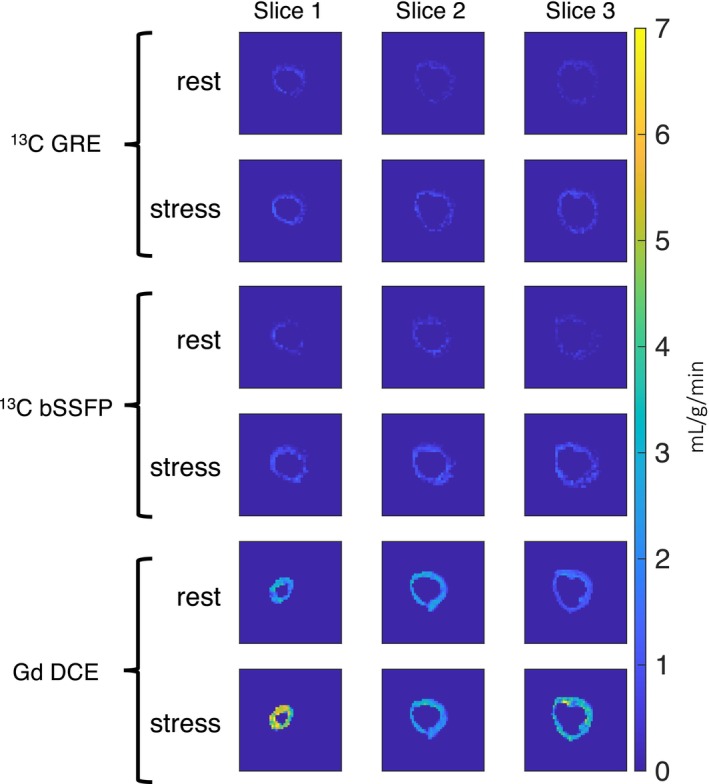
Example quantitative perfusion maps of 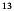
C GRE/bSSFP and dynamic contrast‐enhanced (DCE) from a pig experiment. Slices with the same number are from identical slice locations but acquired at different cardiac phases. The perfusion map from DCE exhibits superior image quality compared to 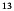
C perfusion maps. Overall, all methods demonstrate higher stress perfusion values than at rest.

Figure 7 displays perfusion maps obtained through 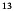
C and DCE‐MRI in a pig experiment. Three slices from the apex (slice 1), middle (slice 2), and base (slice 3) of the heart are presented for each imaging method. Notably, the DCE perfusion map exhibits superior image quality compared to the 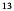
C perfusion maps, attributed to the higher myocardium SNR in DCE compared to 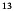
C (Figure 5B). Both 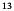
C and DCE demonstrate higher stress perfusion values than in rest. However, generally, the perfusion values estimated by DCE are higher than those obtained from 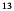
C imaging. 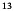
C rest perfusion maps are noisier and have lower quality compared to the corresponding stress maps, primarily due to lower myocardial perfusion signals during rest compared to stress.

Table 1 presents the 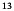
C GRE/bSSFP and DCE estimated mean myocardial perfusion values for each pig, along with the overall mean and standard deviation across the three pigs. The data show that the mean perfusion values are similar across pigs, with a relatively small standard deviation. The 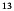
C estimated perfusion values are lower compared to those obtained with Gd DCE for all three pigs. When comparing the 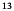
C methods, GRE yields higher mean perfusion values (rest/stress: 0.2/0.5 mL/g/min) than bSSFP (rest/stress: 0.1/0.3 mL/g/min).

**TABLE 1:** The rest and stress myocardial perfusion values obtained using C GRE/bSSFP and dynamic contrast‐enhanced (DCE) methods for each individual pig, calculated as the mean within the myocardial mask.

Perfusion		Pig 1 mean	Pig 2 mean	Pig 3 mean	Mean over pigs	Standard Deviation
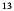 C GRE	Rest	0.2	0.2	0.2	0.2	0.0
	Stress	0.5	0.4	0.5	0.5	0.1
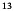 C bSSFP	Rest		0.1	0.1	0.1	0.0
	Stress	0.3	0.4	0.1	0.3	0.1
Gd DCE	Rest	1.7	1.4	2.8	2.0	0.7
	Stress	3.0	2.7	2.8	2.8	0.2

*Notes*: The table also includes the overall mean and standard deviation of perfusion values across the three pigs. All values are reported in units of mL/g/min. The rest perfusion value for pig 1 using 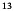
C bSSFP is not available due to a scan failure.

**FIGURE 8:**
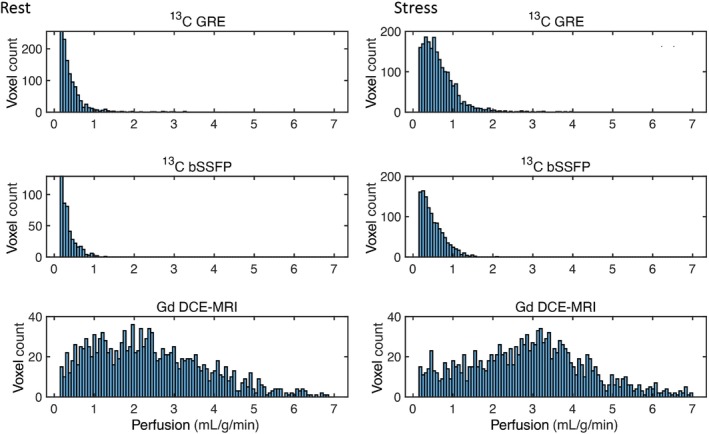
Histograms of myocardial perfusion values at rest (left) and stress (right) obtained using 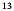
C GRE/bSSFP and dynamic contrast‐enhanced (DCE‐MRI) in three pigs. In both 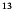
C and DCE‐MRI, stress perfusion values were higher than rest values, as expected. DCE‐MRI histograms show higher perfusion values compared to 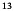
C in both rest and stress conditions.

Figure 8 displays histograms of myocardial perfusion values estimated by 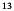
C GRE, 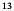
C bSSFP, and DCE for the three pigs. Both 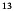
C GRE and bSSFP histograms exhibit similar shapes, whereas the DCE histogram has a distinctly different shape with more high estimated perfusion values compared to the 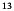
C methods.

Compared to literature perfusion values from PET (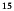
O water) in pigs (approximately 1.5/4.5 mL/g/min for rest/stress conditions^33^), both DCE and 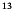
C imaging techniques tend to underestimate myocardial perfusion. However, the perfusion values estimated using DCE are closer to the literature values than those obtained from 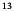
C measurements.

Table 2 presents the estimated MPRI values, calculated as the ratio of mean stress to rest perfusion values shown in Table 1. The table reveals that the MPRI values estimated by 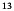
C GRE and bSSFP are similar (approximately 2). The MPRI values estimated using 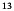
C are closer to the literature value of approximately 3^33^ compared to those estimated by DCE.

**TABLE 2:** MPRI values for each pig measured using the three imaging techniques: C GRE, C bSSFP, and dynamic contrast‐enhanced (DCE).

MPRI	Pig 1	Pig 2	Pig 3	Mean over pigs	SD
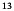 C GRE	2.5	2.0	2.5	2.3	0.3
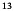 C bSSFP		3.0	1.0	2.0	1.4
Gd DCE	1.8	1.9	1.0	1.6	0.5

*Notes*: The values are calculated as the ratio of mean stress and rest perfusion for each pig. Additionally, the table includes the overall mean and SD of myocardial perfusion reserve index (MPRI) values across the three pigs. The MPRI for pig 1 using 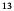
C bSSFP is not available due to the rest scan failure.

## 4 DISCUSSION

### 4.1 Sequence design and simulation

GRE and bSSFP are two widely used sequences in hyperpolarized MRI. We designed both GRE and bSSFP sequences and optimized the sequence parameters to make them suitable for measuring myocardial perfusion. Both sequences were designed to have the same image resolution. However, due to different PSF shapes, the actual resolution varied between the two sequences. bSSFP was two‐times accelerated, and its FOV in the stacked direction was half of GRE's due to its relatively slow encoding speed compared to GRE. The PSF along the x‐ and y‐axes of bSSFP has more pronounced side bands than GRE because of the in‐plane undersampling. However, these pronounced side bands were reduced using conjugate gradient SENSE reconstruction. Variants of the spiral trajectory may improve the PSF, SNR or view‐sharing properties.^34^

The bSSFP sequence was simulated for hyperpolarized MRI, and the results were reported. Based on the simulation results, increasing off‐resonance leads to increased signal oscillation and decay. To increase image quality, the shimming in the imaging region can be optimized. However, shimming of the heart is challenging due to cardiac motion. Another way to reduce signal oscillation is to increase the number of ramp‐up and flip‐back pulses. The simulation shows that ten ramp‐up and flip‐back pulses provide relatively stable signal evolution within a 30‐Hz off‐resonance range.

### 4.2 Coil sensitivity mapping and image reconstruction

The 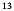
C coil sensitivity mapping is challenging due to the lack of a measurable 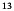
C thermal signal in vivo. This study demonstrates the feasibility of measuring flexible 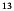
C coil sensitivity maps using the proposed phantom experiment protocol and the designed fixation mechanism. Subsequently, these measured maps were registered to the in vivo experiment based on the scanner coordinates and utilized in the image reconstruction process. The image reconstructed using the measured sensitivity maps exhibits decent quality and moderate SNR. The conjugate gradient SENSE reconstruction method significantly reduced aliasing artifacts in the bSSFP images, contributing to enhanced image quality.

### 4.3 In vivo experiment

The 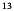
C GRE/bSSFP and DCE images presented in the study demonstrated good image quality, with 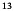
C GRE and DCE images being free of artifacts. Figure 5B revealed that 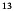
C images exhibit higher SNR in the right ventricle but lower SNR in the myocardium compared to DCE images. This indicates that the 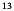
C signal is initially higher than the DCE signal upon reaching the right ventricle. However, due to the fast hyperpolarized signal decay, the 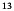
C signal is diminished compared to the DCE signal when the substrate arrives in the myocardium. To improve the 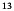
C myocardial signal, optimization of imaging procedures, such as initiating imaging at the onset of left ventricle inflow and increasing the flip angle, can be considered. In Figure 6, it is evident that 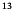
C bSSFP images display superior myocardium contrast compared to 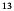
C GRE. This superior contrast in bSSFP may stem from its better utilization of the nonrecoverable hyperpolarized magnetization compared to GRE. DCE perfusion maps exhibit superior quality compared to 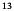
C perfusion maps, primarily due to the higher SNR of the myocardium in DCE images.

Another point to discuss is the effect of gradient infidelity, B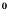 inhomogeneity, and T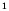/T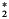 relaxation on perfusion estimation. The expected main gradient imperfection in the scanner is gradient delay. Measured gradient delay times specific to MNS Research Pack are used for correction. For spiral trajectories, gradient delays cause image rotation and blurring. Image rotation does not impact perfusion estimation but may compromise the possibility of spatial alignment. B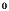 inhomogeneity can also cause signal loss from intravoxel dephasing and related blurring from spiral imaging artifacts, both of which can be estimated. Intravoxel dephasing is characterized by the T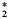 relaxation time. The typical T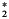 time constant for 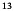
C pyruvate in human myocardium is around 100 ms^35^ for coarse spatial resolution. While no T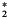 value for HP001 has been reported, it is expected to be similar to pyruvate's T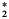, because the T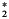 is primarily influenced by field inhomogeneity rather than the long T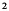 relaxation time. The readout times for the GRE (23 ms) and bSSFP (3 ms) sequences are much shorter than the expected T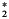 time constant, making intravoxel dephasing effects insignificant.

The severity of spiral imaging artifacts can be estimated from the product of readout time and off resonance in Hz, $t_{\text{readout}}\cdot \Delta f$. For in vivo experiments, the field inhomogeneity in the heart region was measured to result in around ±10 Hz variation of the 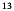
C frequency. For the GRE sequence with a 10 Hz field variation, the resulting dephasing is 72 degrees, leading to blurring. However, the local field variation is much smaller than 10 Hz, resulting in only insignificant off‐resonance effects for the in vivo experiment, which is consistent with the image appearance.

Lastly, T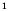 relaxation and RF pulsing can cause image blurring, but the effect is very limited because the T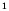 relaxation time of HP001 (37 s in vivo) is relatively long compared to the single image acquisition time (around 400 ms) and small number of excitations (10) applied in a single k‐space acquisition. The resulting image blurring can increase the partial volume effect but the constrained decomposition method used for perfusion quantification is relatively insensitive by design.

The effect of T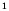 relaxation on perfusion estimation has been studied in a previous paper,^36^ and the T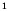 relaxation effect can be incorporated into a one‐compartment perfusion model and mathematically described as the residue function weighted by an exponential decay function with the apparent T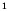 relaxation time in tissue (modified by RF pulsing) as the time constant, denoted as $T_{1}^{\prime }$:

$$C_{t}(t)=F\int_{0}^{t}C_{a}(\tau )R(\tau )e^{-\frac{\tau }{T_{1}^{\prime }}}d\tau .$$

The perfusion F is the first point of the estimated relaxation‐weighted residue function and therefore not affected by the apparent T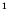 relaxation.

The analysis of estimated perfusion values from three pigs reveals that both 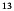
C imaging and DCE underestimate perfusion values compared to literature values obtained from PET (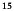
O water). This is likely due to the characteristics of the contrast agents. Water, with its small molecular size and neutral electrical charge, can freely diffuse into tissues, while larger molecules like HP001 and Gd‐based contrast agents cannot easily cross vessel walls and reach the tissue. They require active transport to pass through the vessel wall and reach the tissue, resulting in lower perfusion values compared to PET (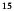
O water). Comparing the estimated perfusion values derived from 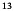
C and DCE imaging, the 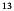
C values are lower. Several factors may contribute to this difference. 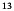
C and Gd‐based contrast agents differ in molecular size and cell permeability, leading to distinct hemodynamics. Consequently, the measured perfusion values from these agents are not expected to be the same. For rest perfusion measured by 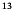
C, the SNR of myocardial tissue is relatively low, which poses challenges for accurately estimating perfusion. Additionally, delays between the left ventricle signal (used as arterial input function) and the perfusion‐weighted signal, in averages 1.5 s in healthy pigs,^37^ can cause approximately 13% perfusion underestimation according to simulations.^30^ Further investigation is needed to fully understand the factors causing perfusion underestimation by 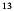
C imaging.

In the case of MPRI estimation, the 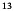
C estimated MPRI values (GRE/bSSFP: 2.3±0.3/2.0±1.4) are closer to the literature value of around 3 compared to the DCE estimated MPRI value (1.6±0.5). The 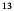
C estimated perfusion changes are consistent with the literature MPRI but since spatial alignment is not an option, the potential scaling of the estimated perfusion values compared to literature cannot be confirmed. Additionally, the reported MPRI values from individual pigs are compared to the literature MPRI value, but physiological variation exists.

## 5 CONCLUSION

The study demonstrated the feasibility of conducting 3D whole‐heart myocardial perfusion quantification using hyperpolarized HP001 with both GRE and bSSFP sequences. However, both 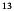
C GRE and bSSFP sequences showed perfusion underestimation compared to the PET (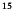
O water) literature value. Notably, GRE exhibited a smaller degree of underestimation compared to bSSFP. In case of MPRI estimation, the 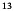
C estimated MPRI values are closer to the literature value than the DCE estimated MPRI value. These preliminary results indicated that 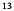
C imaging may more accurately estimate MPRI values compared to DCE‐MRI.

## CONFLICTS OF INTEREST STATEMENT

Jan Henrik Ardenkjær‐Larsen is the owner of Polarize ApS, Copenhagen, Denmark.
